# A typology of community and stakeholder engagement based on documented examples in the field of novel vector control

**DOI:** 10.1371/journal.pntd.0007863

**Published:** 2019-11-25

**Authors:** Cynthia E. Schairer, Riley Taitingfong, Omar S. Akbari, Cinnamon S. Bloss

**Affiliations:** 1 Department of Psychiatry, School of Medicine, University of California, San Diego, La Jolla, CA, United States of America; 2 Department of Family Medicine and Public Health, School of Medicine, University of California, San Diego, La Jolla, CA, United States of America; 3 Department of Communication, University of California, San Diego, La Jolla, CA, United States of America; 4 Division of Biological Sciences, University of California, San Diego, La Jolla, CA, United States of America; 5 Tata Institute for Genetics and Society, University of California, San Diego, La Jolla, CA, United States of America; 6 Center for Wireless and Population Health Systems, Calit2, University of California, San Diego La Jolla, CA, United States of America; Centers for Disease Control and Prevention, Puerto Rico, UNITED STATES

## Abstract

**Background:**

Despite broad consensus on the importance of community and stakeholder engagement (CSE) for guiding the development, regulation, field testing, and deployment of emerging vector control technologies (such as genetically engineered insects), the types of activities pursued have varied widely, as have the outcomes. We looked to previous CSE efforts for clarity about appropriate methods and goals. Our analysis yielded a typology of CSE, and related vocabulary, that describes distinctions that funders, organizers, and scholars should make when proposing or evaluating CSE.

**Methods:**

We compiled available formal documentation of CSE projects, starting with projects mentioned in interviews with 17 key informants. Major features of these examples, including the initiators, target groups, timing, goals, and methods were identified using qualitative coding. Based on these examples, subcategories were developed for a subset of features and applied to the identified cases of CSE in the documents. Co-occurrence of subcategorized features was examined for patterns.

**Results:**

We identified 14 documented examples CSE projects, which were comprised of 28 distinct CSE activities. We found no clear patterns with respect to timing. However, we found that grouping examples according to whether initiators or targets could enact the immediate desired outcome could help to clarify relationships between goals, methods, and targets.

**Conclusion:**

Based on this analysis, we propose a typology that distinguishes three categories of CSE: engagement to inquire –where initiators are empowered to act on information collected through engagement with target groups; engagement to influence –where initiators engage to affect the actions of already-empowered target groups; and engagement to involve –where initiators engage to delegate authority to target groups. The proposed typology can serve as a guide for establishing the goals, identifying appropriate methods, and evaluating and reporting CSE projects by directing attention to important questions to be asked well before determining who to engage and how.

## Author summary

Mosquito borne diseases, such as malaria and dengue, are major causes of illness and death worldwide. Furthermore, it is getting harder to control mosquitoes and other disease-carrying pests because global climate change is facilitating their spread to new areas, and over time, mosquitoes develop resistance to pesticides. Scientists are therefore developing new methods for controlling mosquito vectors using new gene editing tools. However, releasing genetically engineered insects into the environment is controversial. Many experts recommend that communities and stakeholders be consulted about if or how to use these new methods, but there are few guidelines for the best way to do this. We examined published accounts of community and stakeholder engagement pertaining to novel vector control and looked for patterns across these cases. We found that many efforts were not described in published sources, but those that were could be grouped into three categories: engagement to inquire, engagement to influence, and engagement to involve.

## Introduction

Given the increasing global impact of vector borne diseases like malaria and dengue, scientists and vector control professionals are working to replace extant methods such as pesticides with novel vector control methods. For instance, vector control using techniques such as Wolbachia infection of mosquitoes, genetic engineering, and genetic engineering with gene drive may have the potential to cut costs, work more effectively, and avoid exposing humans to the harmful effects of chemicals used in pesticides. However, the release of modified animals, especially genetically engineered organisms (GEOs), is unavoidably political and ethically fraught as outcomes are difficult to predict and could have far-reaching ecological consequences beyond a single nation. Furthermore, tools for national and global science governance and regulation are woefully underpowered to adjudicate the many competing interests and concerns that surround such technology. Determining how to develop GEOs for vector control safely (for humans and ecosystems), responsibly, and ethically requires information and input from outside the conventional domains of science as well as communication across many sectors of society. For these reasons, many have emphasized the importance of community and stakeholder engagement (CSE) as central to resolving how we, as a society, should proceed with these new public health tools [1–8].

CSE in the field of vector control, however, has been an ill-defined concept for which there is a lack of shared understanding regarding appropriate goals, a lack of guidance for selection of CSE strategies, and a lack of evidence supporting the effectiveness of different approaches and established benchmarks for success [7, 9]. Moreover, the many sweeping calls for engagement are vague about how the stated goals of these activities can be met with the methods and resources available. Often, the implication is that any form of engagement will yield desired outcomes such as trust, legitimacy, and acceptance of technology. In theory, engagement is an opportunity for future users to influence technological development and for developers to better understand the public’s interests and make more ethical design choices. In practice, engagement is motivated by strategic needs for information about field testing sites or potential markets, political credibility, or compliance.

As we planned our own project to engage the public on the topic of the emerging technology of gene drive mosquitoes, we found it difficult to articulate how our efforts were similar to or different from other CSE. We wondered if certain types of CSE were more appropriate at different moments in the development of controversial technologies, and we sought a way to determine who should be engaged and how. Here we report on a systematic effort to identify answers to these questions. We examined published examples of CSE surrounding the biological modification of vectors to understand the possibilities and limitations of engagement, as well as inform the design and increase the effectiveness of future CSE efforts in this area. Using a grounded theory approach [10–12], we developed a categorical scheme to organize major features of CSE that has been pursued in the field of novel vector control. Using these categories, we looked for patterns among existing examples of CSE on which to build a taxonomy. Based on our results, we offer a typology of CSE where different types of engagement are categorized according to the intended relationship between initiators and the groups they engage.

### Background

Vector borne diseases, such as malaria and dengue, are major causes of illness and death worldwide [13]. Past attempts to control vectors, such as mosquitoes and mice, with pesticides have had some success at controlling local populations, but at the cost of human exposure to hazardous chemicals. Since the 1950s, scientists have found that populations of insects routinely controlled with insecticides have evolved genetic resistance to these methods [14]. In response to the problems of disease and pesticide resistance, scientists and vector control professionals have sought new methods of controlling pest populations including Wolbachia infection, genetic engineering, and gene drives. Verily and MosquitoMate have teamed up to develop a system to release Wolbachia-infected male mosquitoes that are known to be reproductively incompatible with wild-type mosquitoes as a way to limit mosquito populations. To date, the most well-developed vector control strategy using genetic engineering is Oxitec’s OX513A mosquito. The OX513A is engineered to pass down a tetracycline dependence in the larval stage. Therefore, when OX513A males are continuously released into wild populations these males seek out and mate with wild females. Eggs from females that have mated with OX513A males will hatch but will not survive to grow into adult mosquitoes, thus reducing the total number of biting mosquitoes in the area. Another set of proposals involve creating a “gene drive” that would introduce new genetic traits with preferential inheritance into a wild population. Gene drive could be used to introduce lethal genes that could theoretically eliminate an entire wild population over time. In addition, gene drive potentially could be used to introduce a genetic resistance to disease-causing parasites, like the ones that cause malaria [15, 16], or introduce genetic modifications that would reverse pesticide resistance [17].

Each of these methods promises an alternative to pesticides and may be the key to drastically reducing human suffering and death due to vectored pathogens, but the risks of releasing GEOs into the wild are difficult to determine. These unknowns, coupled with unease regarding the use of genetically engineered crops [18] and the unwanted repercussions of Monsanto’s application of genetically engineered plants in the U.S. agriculture industry [19], have contributed to controversy surrounding the use of GEOs for vector control. Those who fear drastic unintended consequences and those who condemn genetic engineering as “messing with God’s plan” are either opposed to or deeply ambivalent about these technologies [20, 21]. In the face of these unknowns and controversies, observers and stakeholders alike have emphasized the importance of CSE as the technology develops [1–8]. CSE is expected to educate stakeholders or members of the public [2, 5, 22], identify the interests of relevant groups [2, 3, 8, 23], and contribute to site research in preparation for field trials [1, 2, 24]. But many commentators also see CSE as a way to build relationships, trust, and legitimacy for a technology [1, 5, 7, 24, 25], or as a means to more democratic governance by informing or constituting part of the decision-making processes that shape the technology [4–6, 22, 26, 27]. Some also argue that CSE is critical to obtaining community authorization for field trials [4, 28].

This diversity of purpose is reflected in the many activities that fall under the umbrella of “engagement.” While many who call for “public,” “community,” or “stakeholder” engagement have attempted to define it [2, 4, 22], general definitions are unavoidably broad and, therefore, vague. For example, the National Academies of Science, Engineering, and Medicine (NASEM) defined engagement as “Seeking and facilitating the sharing and exchange of knowledge, perspectives, and preferences between or among groups who often have differences in expertise, power, and values.” [2, Chapter 9, p.2]. The World Health Organization (WHO) defines community engagement as “Practices undertaken to inform stakeholders about the diseases and vectors of interest and goals of a proposed research study or intervention trial, and to understand their perspectives and reaction” [22, glossary]. Both of these definitions leave open the question of what kind of “facilitation” or “practices” should count as engagement and how to identify the appropriate “groups” or “stakeholders.”

These slippery definitions are a problem for those who are tasked with advocating for, designing, conducting, and evaluating CSE initiatives. In particular, the slippery definition of CSE was a problem for our team because we were tasked with carrying out public engagement for a technology that was in the very early stages of development. We sought to carefully consider what would count as CSE, who should be engaged, and how.

Lavery [9] recently argued for an evidence base to support good design and practice of CSE and identified the lack of agreed-upon terminology as one obstacle to creating knowledge about CSE. Rowe and Frewer [29] constructed one of the only typologies of “public engagement mechanisms” by focusing specifically on methods of public engagement and classifying them according to the form and flow of information between “sponsors” and “participants.” Though useful and instructive for sorting out already selected public engagement methods, their scheme offers little insight into how best to choose among methods, identify relevant participants, and articulate desired outcomes for projects in development.

We hypothesized that CSE would look different at different stages of technological development and hoped to pinpoint what these differences might be. However, we found no clear patterns with respect to timing. We also found an extreme lack of clarity and communication in the field about what to do and why. Rather than creating a taxonomy that flowed from stage of technological development, we found it necessary to delineate CSE according to desired outcome. Where Rowe and Frewer focused on classifying the *methods* of information exchange, our classification focuses on the desired *outcomes* of engagement. Rowe and Frewer’s taxonomy is difficult to apply before the activities are planned and target groups are identified. In contrast, our typology directs attention to important questions to be asked well before target groups and methods are decided on. Our classification offers a framework and vocabulary that calls attention to how engagement may meet stated goals and, we hope, will encourage funders and critics to be more specific in their calls for CSE by clarifying what the goals should be before deciding who should be engaged and by what methods.

## Methods

### Ethics statement

The research presented here is based on publicly available documents and did not involve human subjects research.

### Overview and scope

We developed a grounded typology following the method of grounded theory [10–12]. We began with a widely inclusive definition of “community and stakeholder engagement” [9] where “communities and stakeholders” could be any persons or organizations with an interest in the outcome of eventual technological deployment and “engagement” could be any form of communication or collaboration between such stakeholders. We focused on documented examples relevant to our work on engagement anticipating the use of gene drives for mosquito control. Using the available documents, we coded the descriptions of major features that might prove central to a definition of CSE. We compared the specific examples of each feature to identify major similarities that could form the basis for subcategorization. Once these categories were developed for each feature, we coded the instances of CSE and looked for broader patterns that could form the basis of a typology.

### Case selection

Systematic reviews often rely on indexed published materials in academic databases. This approach would have been overly exclusive as many of the examples we heard about in our preliminary research were not documented in these ways. We therefore pursued our sample of documents using a method that resembled a “snowball” sample often used in qualitative studies to recruit human volunteers. In preparation for our study of public responses to genetically engineered mosquitoes, we spoke to 17 key informants including public health and vector control professionals, regulatory professionals, and scientists. We began by searching for documentation of CSE efforts mentioned by key informants. We then looked for references to other efforts in the documentation we found. All considered efforts are listed in Table 1.

**Table 1:** Examples included and excluded from analysis.

Included Examples	Excluded Examples (by Rationale)
1. Caged Field Trials in Mexico [30]	*No Comprehensive Report Available*
2. Eliminate Dengue/World Mosquito Project [31–34]	15. Hawaii Focus Groups
3. FNIH Working Group Series [1, 35–40]	16. Landscape Analysis for Gene Drive Rodents
4. Gene Drive Outreach Network [41]	17. Mosquito Mate US Trials
5. Los Angeles 2016 Community Engagement Workshops [42]	18. Oxitec in Grand Cayman [43, 44]
6. Marshall Interviews in Africa [45] [46]	19. Target Malaria [26]
7. Mice Against Ticks [47, 48]	20. UN CBD Forums [49]
8. Mosquito-Free Hawaii 2016 Workshop [50]	21. Verily Singapore Trials [51, 52]
9. NASEM 2015 Workshop [2]	22. Verily US Trials (Debug Fresno) [51, 53]
10. NCSU 2016 Expert Workshop [5]	23. Wolbachia Trials in China
11. Oxitec in Brazil [54, 55]	*No Specific Example Reported*
12. Oxitec in Malaysia [56, 57]	24. CPeace and Island Conservation [58, 59]
13. Oxitec in the US [60, 61]	25. Emerging Ag Inc. [62]
14. Venter Institute 2016 Workshop [24, 63]	26. Public Research and Regulation Initiative [64]
	*Not Clearly Related to CSE*
	27. Pew Trust Report “Bugs in the System” [65]

We considered any example that included the education, outreach, solicitation of information, or other organized interaction with stakeholders outside the initiating organization. Examples also had to be relevant to mosquito control with gene drives, including novel mosquito control and vector control involving genetic engineering. We excluded examples for which references to CSE activities were not specific, or where only engagement materials, brief descriptions, press coverage, or conference presentations could be accessed but for which formal documentation was not available. We also excluded examples that were not clearly oriented toward novel vector control or were not clearly related to CSE.

### Category development

The documents describing the selected examples were loaded into Atlas.ti [66]. RT initially coded text that described the following nine features of CSE that we expected would be easy to identify and relevant to drawing distinctions: (1) “timing” in relation to the development of the technology in question; (2) “initiator” of the CSE; (3) the “motivation” for CSE; (4) the stated “goals”; (5) the “target” group(s); (6) the “method” of engagement; (7) the key “messages” to the target group(s); (8) the “questions” asked of or about the groups; and (9) the stated “conclusions” or recommendations. Based on reading the coded text, CS constructed subcategories for features that were clearly documented for a majority of cases. This process meant that the first round of coding was reviewed by a second researcher.

### Categorization of activities

The documents we collected often discussed multiple engagement activities related to a single case but targeting different groups and sometimes initiated by different organizations. For example, a single document described a series of activities to educate or involve various stakeholders in the testing of Oxitec’s genetically engineered mosquito in Brazil [55]. Because so many of our identified features were particular to the activity, we identified and categorized the specific “activities” defined as a unique target-method dyad.

CS and RT independently applied the developed subcategories to each “activity,” then compared and discussed the results, coming to consensus where there were discrepancies. We compiled a table of distinct activities and their categorized features (S1). Based on these data, we examined patterns of co-occurrence that might inform a logical taxonomic structure for CSE in the field of novel vector control.

### Category iteration

Analysis of the first set of categories suggested that an important dimension of CSE was not captured. After diagraming the logical relations between categories such as goals, initiators, targets, and methods, we noticed that the relationship between initiators and targets with respect to a goal could be characterized as well. We therefore created another set of categories to capture who (initiator or target) began with the power to enact the outcome (as desired by initiators) of the engagement effort and whether this power was delegated in the course of the engagement. These would have been difficult to connect to specific passages in the original texts but could be determined by examining the goals and methods of each activity. We added these to the data table and categorized each activity based on evidence in the texts.

## Results

### Identified cases

**Fig 1:**
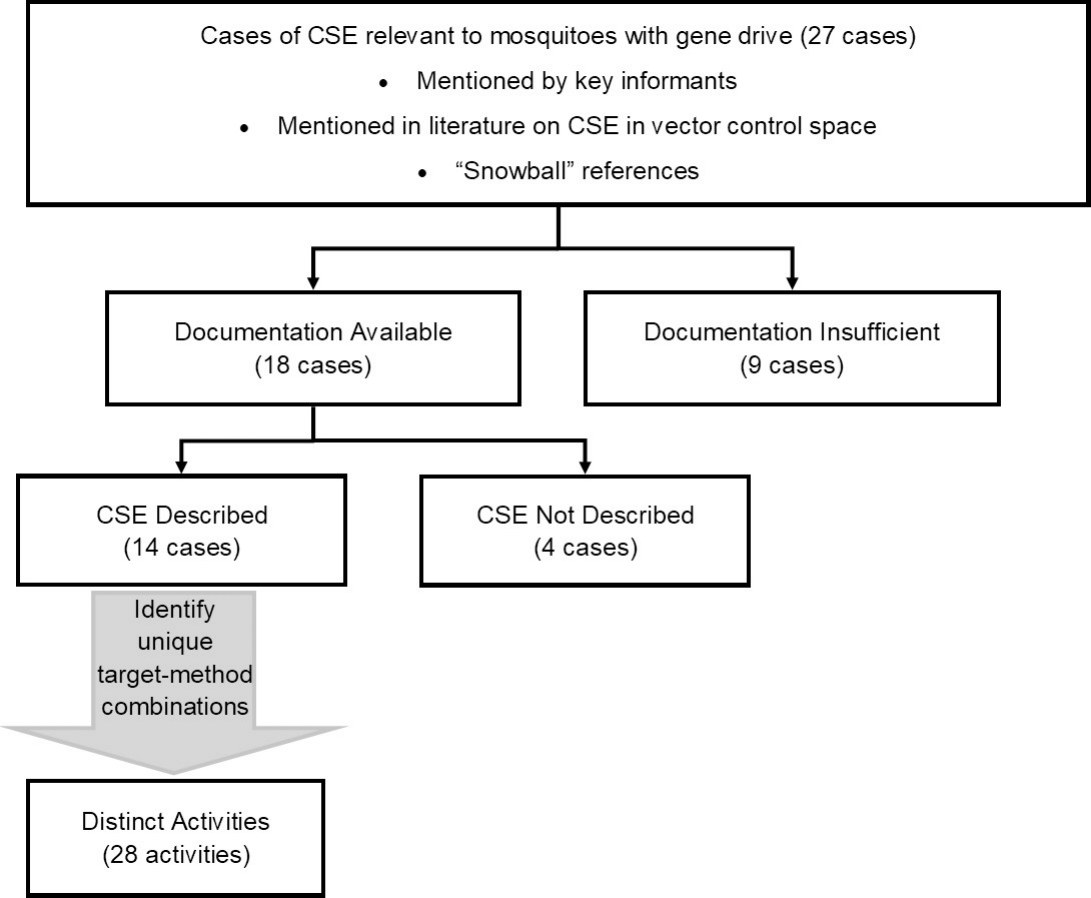
Case selection.

We sought documentation for 27 unique examples of CSE projects (Table 1). We excluded nine examples that were not sufficiently documented, three examples that were not clearly related to novel vector control, and one that was not clearly related to CSE. This left 14 examples of CSE projects oriented toward novel vector control technologies. Fig 1 illustrates this selection process. Table 2 presents the included cases and a short summary for each. The cases we considered revolved around three main methods for biologically modifying vectors: Wolbachia-infected mosquitoes, genetically engineered organisms (GEOs), and genetically engineered organisms with gene drives (GEOs with GD). Aside from an early expert working group in 2008 (an FNIH Working Group) most projects happened between 2010 and 2016 (Mice Against Ticks is ongoing).

**Table 2:** CSE example projects and activities by type of technology.

Example Projects	Activities	Description
1. Caged Field Trials in Mexico	Negotiations with national agencies and local government for authorization	In 2008, a collection of scholars working on new approaches to mosquito control sought a regulatory pathway to hold caged field trials of a strain of *Aedes aeypgti* in southwestern Mexico. This effort involved a range of engagement activities to satisfy community regulatory processes and establish new opportunities for community input, including acquisition of approval from the necessary parties (e.g. research boards, biosafety commission, a local farming collective), presentation of documents at outreach meetings, and public notice and comment, all at the local and national levels.
2. Eliminate Dengue/World Mosquito Project	Face-to-face canvassing, traditional public relations activities, meeting with local leaders, public meetings	Eliminate Dengue (now known as the World Mosquito Program) is a non-profit initiative founded in 2011 in northern Australia to fight mosquito-borne diseases using the Wolbachia method. Their efforts in Queensland, Australia included a combination of “community-facing activities,” entomological release activities, and direct experiments, in which scientists designed additional research projects to address community concerns about the Wolbachia method.
3. FNIH Working Group Series	Expert Working Group	In 2008 and 2016, the Foundation for the National Institutes of Health (FNIH) convened core working groups of scientists to produce recommendations for contained field trials of GE mosquitoes.
4. Gene Drive Outreach Network	Public-facing Website	A group of 8 foundations and organizations dedicated to the prevention of mosquito-borne disease partnered to raise awareness of the value of gene drive research. The website is one of their activities.
5. Los Angeles 2016 Community Engagement Workshops	Community Engagement Workshops	In 2016, the Keystone Policy Center conducted a series of 5 community engagement workshops on behalf of public health agencies in the greater Los Angeles area. The workshops focused on assessing community preferences, values and concerns with various mosquito control techniques.
6. Marshall Interviews in Africa	Interviews and surveys of members of public in Mali and surveys of experts in Nigeria	From 2008–2009, Marshall and colleagues conducted a study of public perception of GE mosquitoes as a tool for malaria control in Mali. In a companion study, the team studied Nigerian scientists’ receptiveness to potential releases of GE mosquitoes for malaria control.
7. Mice Against Ticks	Expert workshop, community meetings, online platform	Mice Against Ticks is a project to develop novel solutions to the threat of Lyme disease in New England. The proposed technology would involve genetically engineering mice that are resistant to hosting Lyme disease and ultimately “driving” this trait into island populations of mice. Mice Against Ticks is also a pilot program for “Responsive Science”–an experimental framework for citizen-driven science.
8. Mosquito-Free Hawaii 2016 Workshop	Expert Workshop	In 2016, 13 institutions, foundations, and agencies convened a group of experts in mosquitoes, mosquito-borne disease, public health, and wildlife for a workshop focused on new approaches to mosquito control and elimination in Hawaii.
9. NASEM 2015 Workshop	Expert committee	The National Academies of Science, Engineering, and Medicine (NASEM) convened an expert committee in 2015 to produce a guidance document for the development of GEOs with gene drive. The report discusses science, ethics, public engagement, and governance related to gene drives.
10. NCSU 2016 Expert Workshop	Expert Workshop (“collaborative policy design”)	In 2016, scholars at North Carolina State University (NCSU) convened a 3-day expert workshop with support from the National Science Foundation (NSF). The workshop brought together more than 70 experts from academia, business, government, and non-profits. A 2018 special issue of the Journal for Responsible Innovation documents presentations and recommendations for governance and research related to gene drives.
11. Oxitec in Brazil	Expert commission, media coverage, contact with government officials and regulators, home and school visits	Since 2009, Oxitec’s OX513A mosquitoes have been used experimentally in Brazil as a tool for dengue control. The Brazilian NGO Moscamed launched *Projeto Aedes Transgênico* (PAT), a partnership between Oxitec and University of São Paulo researchers, that would propose and eventually carry out releases of OX513A throughout Brazil.
12. Oxitec in Malaysia	Public notification and comment, expert workshop, public meetings	The Institute for Medical Research (IMR) in Malaysia partnered with Oxitec to develop a GE mosquito, known as OX513A-My1, for vector control. When IMR sought regulatory approval for a limited open release of the strain in an uninhabited forest, risk assessment and public engagement were conducted.
13. Oxitec in the US	Public notice and comment, public meetings, face-to-face canvassing, traditional public relations activities, surveys, voter referendum	As early as 2009, Oxitec sought U.S. regulatory approval for their OX513A GE mosquito. In 2015, Ernst and colleagues conducted a survey of state residents to assess awareness of the trial. In 2016, the US FDA solicited public comments on its environmental assessment of OX513A. In response to growing controversy, Monroe County Florida added a non-binding referendum to the November 2016 ballot to assess voters support of the trial.
14. Venter Institute 2016 Workshop	Expert Workshop	In 2016, scholars at the J. Craig Venter Institute convened a two-day workshop at UCSD. The workshop included scientists, federal regulators, ecologists, ethicists and environmental policy analysts, as well as experts in lab biosafety, insectary standards and operation, GE insects and other vector control methods. The workshop focused on safety and science policy related to gene drive insects.

Within the documentation for the 14 included cases, we identified descriptions of 28 distinct CSE activities defined by a unique target-method dyad. In addition, we identified descriptions of three risk assessment activities that involved submitting the technology to an independent body for formal risk assessment. However, we have excluded these examples from our analysis on the grounds that the independent bodies were, by definition, not stakeholders in the decision to deploy the technologies they reviewed. This is in contrast to expert workshops in which the participating experts included scientists working to develop the technologies in question.

### Developed categories

Based on coded text, we created subcategories for five of the nine coded categories. Table 3 presents these subcategories as well as the count of activities that fell in each. We discuss each of these features below in more depth. Four features that we initially attempted to code (motivations, messages to the target group(s), questions asked of or about the target groups, and conclusions and recommendations) were difficult to identify, compare, and categorize due to inconsistent or vague reporting. For example, many conclusions and recommendations were difficult to distinguish between broader conclusions of the publication. Features like motivations and messages to target groups were most often not explicitly described in the text.

**Table 3:** Number of activities coded to each feature subcategory. (*indicates where subcategories are not mutually exclusive).

Feature	N of coded activities
Timing	
Research and Development	12
Regulatory	2
Trial	13
Various Technologies	1
Initiators*	
For-profit	4
Non-profit	16
Academic Institution	1
Individual Researchers	5
Government Agencies	7
Targeted groups	
Experts	9
Geographically defined communities	16
Geographically defined leaders	1
General Public	2
Methods	
Expert Workshop	6
Canvassing	3
Public Relations	3
Notification and comment	2
Public meeting	6
Social science	5
Other	3
Stated Goals*	
Compliance	4
Inform	7
Knowledge Production	10
Policy Production	6
Dialogue	6
Intangible	11
Who can act?	
Target	8
Initiator	20
Delegation of power?	
Yes	6
No	22

### Timing

We identified CSE activities that took place during the research and development (R&D) phase (n = 12) where the proof-of-concept was either yet to be developed or in early stages of development, during the process of regulatory approval (n = 2), or surrounding a field trial (n = 13). In the one remaining activity (Mosquito-Free Hawaii Workshop), participants considered and compared multiple technologies at various phases of development. The activities where participants considered mosquitoes and mice with gene drive were undertaken very early–before any viable technology was available–as this technology is still in development. Others, including CSE for Wolbachia infected mosquitoes and Oxitec’s OX513A mosquito happened in preparation for or during field trials. These technological phases are not exhaustive, notably missing post-field trial phases, such as bringing technologies to market. The identified subcategories highlight the uneven documentation of CSE.

### Initiators and targets

While sometimes only implied, initiators and target groups were identifiable for all activities. In some cases, they were described with vague language, such as when “the public” was targeted. Explicitly identified initiators fell into five subcategories (not mutually exclusive): for-profit companies (n = 4), non-profit organizations (n = 17), academic institutions (n = 2), individual academic researchers (n = 5), and government agencies (n = 7). Target groups fell into four main categories: experts (n = 9), geographically defined communities (n = 16), geographically defined leaders (n = 1), and general public (n = 2). Geographically defined communities could be as narrow as the residents of a particular neighborhood or as broad as the citizens of an entire country. Geographically defined leaders are distinct from government agencies in that the former includes individual local and community leaders.

### Methods

The documented methods fell into six categories. Expert workshops (n = 6) included any meeting or conference of expert stakeholders that culminated in the drafting of a group statement. Canvassing (n = 3) included efforts to connect with individual community members and residents face-to-face through door-to-door visits, “walking the streets” [31], or presence at community festivals or events. Public relations (n = 3) included activities such as press releases, advertising, educational campaigns, and branding techniques such as using uniforms, logos, or mascots. Notification and comment (n = 2) included processes where information was distributed to stakeholders or made available to the public and written responses were accepted or solicited. Public meeting (n = 6) included any form of outreach organized as a meeting or lecture open to members of the community, whether or not the meeting was organized through a local governing body. Social science (n = 5) included methods such as interviews, surveys, or various forms of focus groups to identify the relevant interests and concerns of the target groups.

### Goals, who can act, and delegation

Goals were difficult to identify in the text and difficult to characterize, requiring two rounds of subcategorization. Our initial set of codes sought to reflect not only the hoped-for outcome, but the process of getting to that outcome. For example, the organizers of Mice Against Ticks hoped to bring community members into scientific decision-making and the publication documenting the NCSU Expert Workshop described the goal as “collaborative policy design.” In our original schema, we had labeled the goals of such activities as “governance,” but found this category difficult to assign based on the available description of most activities. To make the subcategories easier to apply, we created a new set that reflects the clear near-term goal of the effort. We also developed two other codes to capture the relationship between these goals and the initiators and targets.

We identified six subcategories of goals that are not mutually exclusive:

Activities that aimed for policy production (n = 6) culminated in recommendations for policy or scientific best practices. The NAS Expert Committee, NCSU Expert Workshop, Venter Institute Expert Workshop, and FNIH Working Group Series all aimed at producing statements that set out best practices and guidelines for scientists and other technology developers to refer to for definitions of due diligence in research safety and ethics. These guidelines included technical rules regarding, for example, laboratory safety or procedures for caged field trials, and ethical guidelines about, for example, the importance of engaging other stakeholders as the technology develops.

Activities with a knowledge production goal (n = 10) often took the form of social science projects organized to collect information about or from target groups that can inform, for example, policy or project planning. Documents discussed the importance of identifying and articulating stakeholder interests. This was the primary goal of the Los Angeles 2016 Community Engagement Workshops, Oxitec in Malaysia, Eliminate Dengue, and the Marshall Interviews in Africa. Often these activities aimed to assess knowledge as well as attitudes and concerns. The Mosquito-Free Hawaii workshop was also assigned a knowledge production goal, as the output of the meeting was a white paper, presumably meant to inform members of the funding organizations on the topic of novel vector control.

Activities with a dialogue goal (n = 6) sought to create opportunities for in-person two-way communication between initiators and targets. Canvassing carried out by Eliminate Dengue in Australia and by Moscamed in Brazil as well as public meetings held by Mice Against Ticks were initiated to allow for residents to receive information and ask questions in real time.

Activities with an inform goal (n = 7) were aimed at delivering information or communicating a message to the target groups. Eliminate Dengue, the Gene Drive Outreach Network, Oxitec in the US, Malaysia, and Brazil, and Mice Against Ticks all included activities aimed at informing, even if education was not explicitly stated as a goal. It is also possible that other examples included undocumented informing activities.

Activities with compliance goals (n = 4) were described as meeting a government and funding agency requirement.

Activities with an intangible goal (n = 11) were accompanied by at least one of the other types of goals mentioned above. Intangible goals included hard to measure outcomes such as building trust or positive relationships within a community. Intangible goals were discussed explicitly in sources on Eliminate Dengue and Oxitec in Malaysia. These included gaining “public trust” (Malaysia p. 1323) and doing CSE that is “meaningful to stakeholders” (Eliminate Dengue, p. 7). While we might infer goals such as building trust, relationships, or political accountability for the other examples, such goals were not stated.

In the course of developing these categories, we noticed that goals are associated with desired outcomes that often go without explicit articulation. For example, when researchers conduct a study of public attitudes, their immediate desired outcome is to publish a paper that adds to public knowledge and can inform public health policy and public health intervention deployment. When a non-profit organization initiates a public relations campaign, the immediate desired outcome is greater public awareness and perhaps a change in the public’s behavior. To capture the relationship between initiators or targets to these immediate desired outcomes, we coded each activity according to who had the power to enact the primary outcome wished for by the initiators.

Targets had the power to act when the desired outcome could not be realized without their action or cooperation. This was the case for eight activities in our sample. The clearest examples are when target groups are leaders or government agencies who are charged with granting permission or regulatory approval. For instance, researchers could not conduct caged field trials in Mexico without a favorable vote from members of the farming collective empowered to control the sale of neighboring property. Members of the public can be empowered targets as well. For instance, Moscamed hoped that, in response to its media campaign, members of the Brazilian public would continue to apply their repellent or remove standing water at their homes even after experimental mosquitoes were released.

In contrast, initiators were the empowered actors when they were the ones to take action in response to the CSE. This was the case for 20 activities in our sample. Examples of empowered initiators include the scholars who studied and published on public attitudes in Mali, Nigeria, and Florida; the mosquito control agencies who sponsored the Los Angeles community engagement workshops to guide their policies; and the Eliminate Dengue team who responded to local concerns by designing new experiments.

In most cases, the power to act begins and ends with either initiators or target groups. However, in a few examples the outcome of the engagement is a delegation, sharing, or transfer of power. Therefore, we also coded CSE activities according to whether the power to enact an outcome was transferred in the course of the activity. We coded power as delegated in six of our 28 example activities. For example, when expert working groups who were convened to write and publish statements under the banner of the initiating institution, the authority of the institution was delegated to the target experts who decided on the content of the reports. The example of Mice Against Ticks provides a very different potential example, where the project team aspired to delegate the authority of a principal investigator to the communities who may ultimately use the products of the research. However, it is unclear from the available documentation if or how this was actually achieved. To be a true delegation of power, there would need to be a mechanism for the target community to enact research decisions rather than recommend decisions that investigators could choose to ignore. We ultimately coded this as an example of when initiators retained the power to act, albeit with the good faith intention to follow the recommendation of the community they consulted.

### Relationships between features

Based on the categorized activities, we created various crosstabulations to see the frequency of overlap between features. We sought a way to organize CSE activities into a typology based on features that we expected would be easily identified and relevant. However, we found that no combination of our initially coded features could clearly sort out the diversity and inconsistency among these activities in a sample of this size. There was no clear pattern connecting the timing of CSE to features like initiators, targets, or methods, nor were methods clearly associated with types of target groups.

Table 4 presents the relationships between coded goals and timing, showing too few examples in the regulatory category and substantial overlap between the R&D and trial categories. The relatively small sample size combined with high variability makes a relationship impossible to decipher. In contrast to Table 4, Table 5 illustrates how the data can suggest an expected logical relationship, showing that most goals were pursued with appropriate and expected methods. There were also projects where the stated goals did not clearly match up with the chosen methods. For example, a public meeting where an expert lectures to a crowd may not be the most effective way to promote dialogue between initiators and targets. Similarly, using a process of notification and comment to create knowledge about public attitudes will likely result in a skewed picture of public will.

**Table 4:** Co-occurring timing and goals. (Shading added to highlight most populated cells).

	Timing			
	R&D	Regulatory	Trial	Other
Total	12	2	13	1
**Goals**				
Compliance		2	2	
Dialogue	1		5	
Inform	2		5	
Knowledge Production	3	1	5	1
Policy Production	6			
Intangibles	4		7	

**Table 5:** Co-occurring methods and goals. (Shading added to highlight most populated cells).

	Method						
	Public meeting	Notification & comment	Expert Workshop	Canvassing	Public Relations	Social Science	Other
Total	6	2	6	3	3	5	3
**Goals**							
Compliance	1	2			1		
Dialogue	3			3			
Inform	1	1		1	3		1
Knowledge Production	1	2	1			5	1
Policy Production			5				1
Intangibles	4	1		3	1	1	1

The empowered actor categorization did appear to have a relationship to both goals and methods, as illustrated in Table 6. Most of the activities with an empowered initiator were associated with dialogue, knowledge production, or intangible goals as well as with public meeting or social science methods. Most of the activities with empowered targets were associated with inform and intangible goals and with public relations methods. Nearly all of the activities with power delegated to targets were associated with policy production goals and all used expert workshop methods.

**Table 6:** Empowered actors co-occurring with goals and methods. (Shading added to highlight most populated cells).

	Empowered Actor		
	Initiator	Target	Target (by delegation)
Total	14	8	6
**Goals**			
Compliance	1	3	
Dialogue	5	1	
Inform	0	7	
Knowledge Production	8	1	1
Policy Production	1		5
Intangibles	7	4	
**Methods**			
Expert Workshop			6
Canvassing	2	1	
PR		3	
Public Meeting	4	2	
Social Science	5		
Notification and Comment	1	1	
Other		1	

## Discussion

The relationships between the categorized features gives us a sense of the landscape of CSE in the field of novel vector control. First, this analysis highlights the need for better, more consistent reporting of CSE efforts in this field. While there may be optimal strategies for who to engage and how at different moments in the technological development of mosquito control, these are not identifiable from the available information. Sufficient documentation was only available for half of the projects we were aware of, and the documentation we included was extremely inconsistent in the level of detail provided. Due to a lack of formal or comprehensive documentation, we excluded important examples such as Target Malaria and Debug Fresno. Often even when formal or academic documentation was available, it was difficult to identify specifics about who was involved and what was done. The general lack of documentation of so many examples of CSE makes it very difficult to learn from past efforts and underscores the need for the field as a whole to adopt standards and best practices for dissemination of CSE initiatives in novel vector control. Given the importance and expense of CSE, standards for dissemination would support better understanding, planning, and improvement of future projects.

Second, we found that many projects hoped to achieve a variety of goals through CSE, some of which were not clearly connected to the chosen method. This was especially true of the difficult-to-measure goals we coded as intangible, such as building public trust or accountability. Simply initiating an activity and calling it “public engagement” is not necessarily enough to meet such goals.

Third, our analysis of empowered actors illuminates the sorts of relationships that are enacted through CSE in this field. While commentators’ calls for CSE often imply that these activities are meant to empower and involve stakeholders who would otherwise be left out of decision-making about emerging technology, our analysis suggests a more nuanced view of CSE. When we look at how the results of CSE are likely to be used and by whom, we see that the majority of documented CSE activities provide the initiators with information that they will then publish, consider, or, later, share with other already-empowered actors such as legislators, local leaders, or voters. In fact, where targets are the empowered actors, the information that is disseminated likely reflects findings from engagement with experts, leaders, or community members. While public relations campaigns may not be considered paradigmatic CSE, these activities illustrate the importance of informing those who already have the power to act, even if the desired action is acceptance or compliance. Finally, by including cases where experts were engaged and offered the chance to publish their recommendations under the banner of reputable organizations, we found unexpected examples of delegated power. While our identified examples are somewhat homogeneous, they can serve as a model for what might be required of a CSE strategy that truly redistributes decision-making power.

Although CSE activities must be tailored to specific contexts, clarification of how engagement relates to decision-making would go a long way toward making the notion of CSE both more meaningful and useful. Based on our analysis, we suggest a typology that is applicable to the field of novel vector control but also to other areas of CSE. The typology names three types of CSE identified by who holds the power to enact the immediate desired outcome: 1) engagement to inquire; 2) engagement to influence; and 3) engagement to involve.

Engagement to inquire takes place when initiators reach out to learn something about or from the target group. Engagement to inquire does not imply that initiators are required to act on the gleaned information in any specific way. The existing system of notification and comment in the U.S. regulatory system is a form of engagement to inquire because notification and comment supplies regulators with information they value in making their rulings, but they are not required to change their ruling based on public comments. When Monroe County, Florida added a non-binding referendum to the 2016 ballot about the acceptability of hosting field trials of Oxitec’s genetically engineered mosquitoes [67], this was also a form of engagement to inquire. Neither the county, nor the mosquito control district was legally obligated to take the results into account, even if that was their intention. In the case of academic studies, the investigator who engages to learn about a group ultimately makes the decision of whether, when, what, and where to publish, and such findings may never influence technology development or policy decisions.

Engagement to influence includes any effort to educate or deliver a message to target groups with the intention of influencing decisions, inspiring action, or changing behaviors. There are many forms of engagement to influence, from public health campaigns to lobbying Congress. It is widely practiced by government agencies, non-profit organizations, and for-profit companies. When the U.S.-based team sought permission for caged field trials in Mexico, they had to engage with both Mexican officials and a farming collective. These activities should be classified as engagement to influence because the engagement served to educate these groups about the project and to encourage them to authorize the trial under their existing jurisdiction.

Marketing and public relations professionals have perfected engagement to influence and it is often powerful and highly effective. However, the political implications of engagement to influence must be taken into account, especially when intangibles like trust are desired. Under some circumstances engagement to influence can get the word out about something the target groups are inclined to support or trust or be seen as the accepted standard within a legislative process. Under other circumstances it can be challenged as a ploy to influence democratic politics or scientific decision-making. When the scientists of Eliminate Dengue made public presentations to communities in Queensland Australia, they helped to show their commitment to keeping the community informed. By the account provided by Kolopack et al, this was effective in building and maintaining trust. Similarly, public relations campaigns in Brazil helped locals understand why mosquitoes were being released in their neighborhoods to mitigate surprise or fear that might lead to protest. However, similar strategies employed in the Florida Keys in anticipation of field trials did not have this intended effect. Instead, the campaign alerted and fueled an opposition movement that challenged many official messages and worked to undermine trust in the local mosquito control authorities.

Engagement to involve implies that a decision-making process is opened to members of a target group that would otherwise be outside their sphere of influence. Power held by the initiators is offered and shared to the target group being engaged. When academic institutions convene a group of experts to construct guidelines for the safe conduct of gene drive research, the institution is offering those experts the power of a platform from which they can produce a highly influential, credible, and/or legitimate document. In theory, when Mice Against Ticks invites residents of Martha’s Vineyard to design a research program, the science team offers to share the power of the principal investigator to guide the research in the laboratory. Though some might consider this to be the ideal form of CSE, engagement to involve was the least common compared with the other two types of CSE.

**Fig 2:**
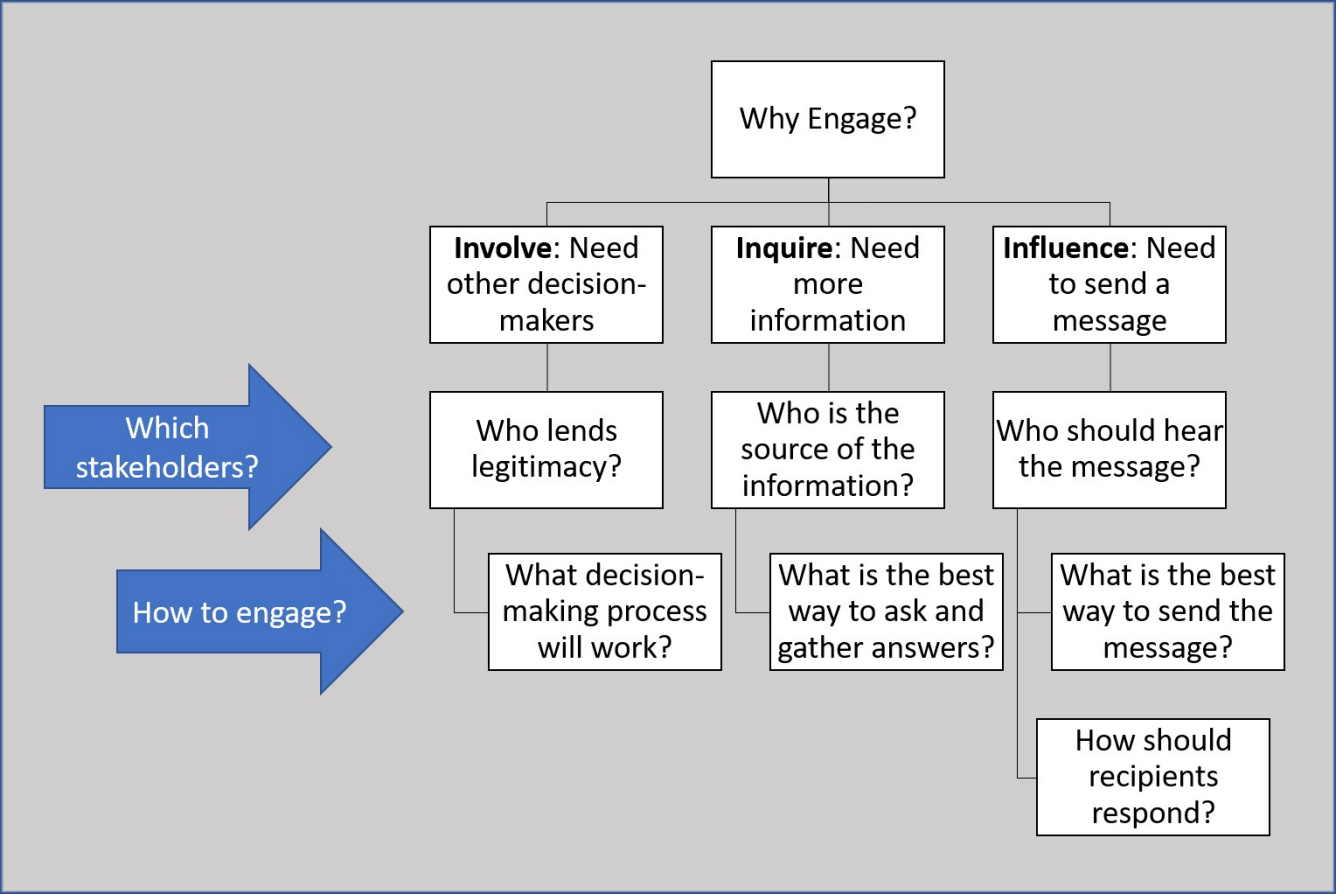
Application of typology when planning CSE efforts.

Fig 2 illustrates how our typology can be the starting point for identifying the appropriate type of CSE, the appropriate target groups, and the appropriate methods for achieving a certain goal. When calling for, funding, or organizing an effort to engage, the first question that must be answered is “why?” The answer must go beyond stating vague intangible goals to specifying how engagement is the appropriate path to more informed decision-making, legitimacy, trust, or improved relations. Is there a need for more information? Is there a need to get a message out and win hearts and minds? Or, is there a need to share decision-making power? The answer to the question of why will then inform choices about who to engage and how. Who has the needed information or who needs to be studied to create the needed information? Who needs to hear the message and what should they do when they hear it? Who needs to be involved in decision-making to render those decisions more legitimate? Only after these questions are answered, can appropriate methods for engagement be selected, developed, initiated, and evaluated.

### Limitations

The scope of this analysis was limited by the available written accounts of CSE in the field of novel vector control, which underscores the extent to which CSE in this field is grossly under-reported in the literature and the need for greater dissemination. For instance, we excluded known CSE projects that were not formally documented. In addition, the available sources were diverse and, in many cases, did not contain enough information to answer basic questions about how the CSE activities were carried out and why. Because our typology grew out of this limited set of examples, it is possible that there are other types of CSE for novel vector control that we have not considered. There are forms of CSE we are aware of, such as marketing activities or lobbying, that were not represented in our sources. However, as discussed below, our classification can be readily applied to these types of CSE and to forms of CSE in other fields.

### Conclusion

Growing concern about the spread of vector-borne diseases has prompted the development of novel vector control technologies that rely on the biological modification of vector organisms. However, the development, testing, and deployment of these tools is a complex process with potentially far-reaching consequences. Therefore, many in the field of vector control agree on the importance of CSE. However, CSE in this field has been a previously ill-defined concept for which there is little shared understanding. There is a great need for guidance regarding appropriate goals and selection of CSE strategies as well as evidence supporting the effectiveness of different approaches and established benchmarks for success. We analyzed the best-documented examples of recent engagement in the development of novel vector control technologies and catalogued the features of a wide variety of efforts that fall under a general definition of CSE.

CSE is often called for as a form of decision-making [4–6, 22, 26, 27], but the suggested typology clarifies why this is problematic. Engagement to inquire and engagement to influence each may inform decision-making by providing information to empowered decision-makers, but the CSE itself is not a decision-making process. Because engagement to involve is defined by a sharing or transfer of power, it cannot replace an existing political process unless that political process allows for such replacement. For example, critics might argue that U.S. regulatory agencies should allow for members of the public to be part of regulatory decision-making, but without legislative change, at least in the U.S. context, no amount of engagement can make this so.

A vocabulary that differentiates between these types of engagement is a critical tool for those who believe that engagement should be central to the development of potentially controversial technologies to address vector control. Defining engagement with reference to the power to act will 1) help initiators identify their goals, for themselves, so that they may design and conduct their engagement activities in the most appropriate and useful ways; 2) Help initiators to better and more transparently clarify and communicate their goals to the target groups they engage; and 3) Help those target groups be informed about their role in (and the overall purpose of) a given engagement activity.

Without this work, projects that set out to involve easily may be susceptible to evolving into projects to inquire or to influence because engagement to involve requires initiators to give up some amount of power and work to find an appropriate way to structure the involvement of target groups. For the same reasons, projects that inquire or influence may erroneously be touted as efforts to involve, which may come to be seen as disingenuous attempts to co-opt target groups. In principle, any type of engagement can generate positive relationships between initiators and target groups, but this depends, in part, on setting shared expectations through transparency and clarity of purpose.

## Supporting information

S1 Datasheet Collected details of included CSE activities. Notes and codes assigned to individual activities for each analytical category discussed in this publication. Codes are highlighted when discussion was required to reach consensus about appropriate coding.
(XLSX)
